# Supplementary figures and images for: Replacing critical point drying with hexamethyldisilazane drying enhances the ultrastructural preservation of cell surface projections in the parasite Trichomonas vaginalis for scanning electron microscopy
Source: PLoS One. 2025 Oct 6;20(10):e0333745. doi: 10.1371/journal.pone.0333745 (PMC12500116; doi:10.1371/journal.pone.0333745)

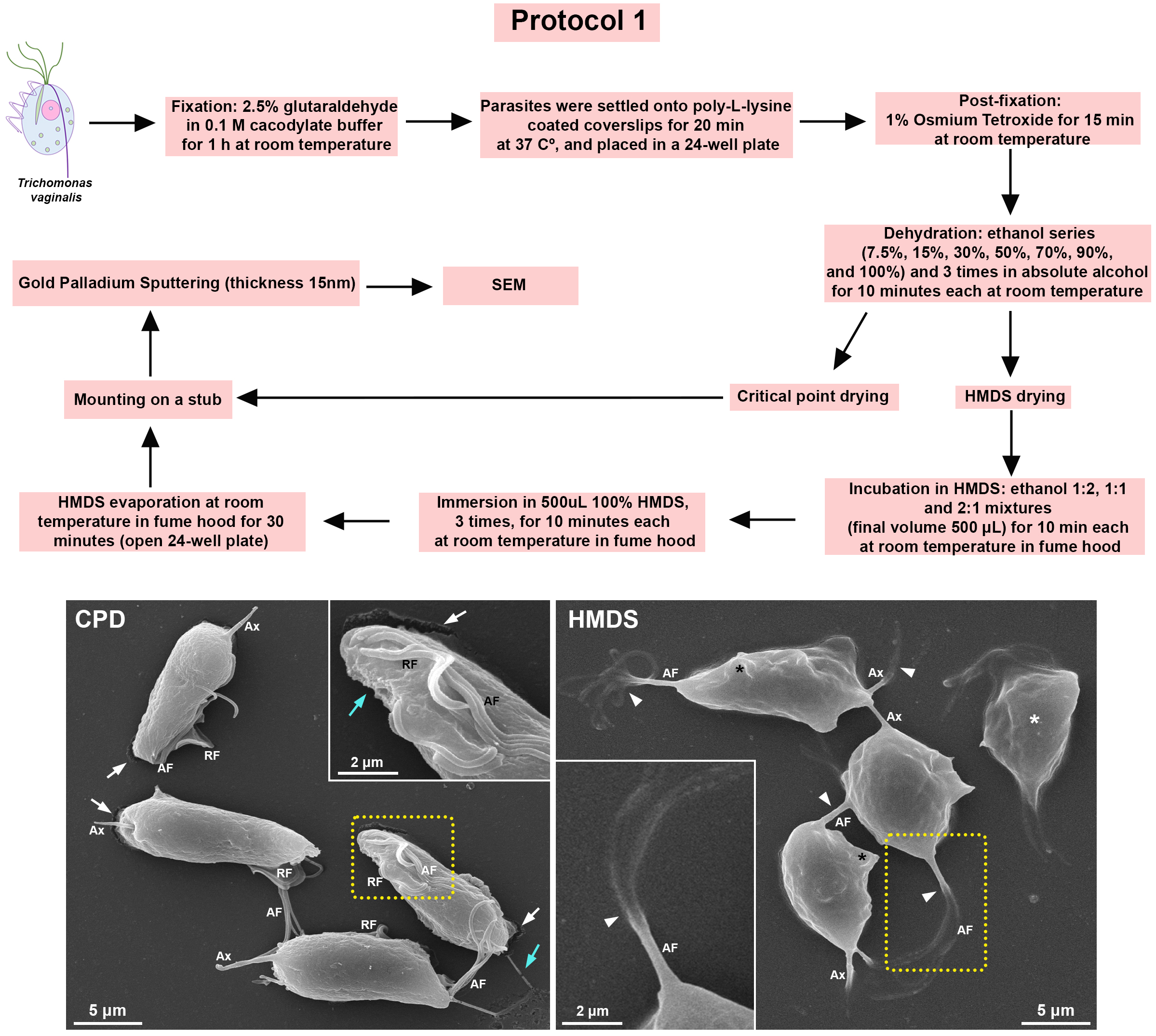

Supplement: S1 Fig — In CPD-prepared samples, parasites exhibit typical piriform or ellipsoid morphology with a slightly irregular surface displaying small undulations. Four anterior flagella (AF), a recurrent flagellum (RF), and the axostyle (Ax) tip extending from the posterior region of the parasite are seen. Artifacts such as cell shrinkage (white arrows) and ruptured structures (blue arrows) are also observed. In HMDS-prepared samples, parasites display a flattened surface; anterior flagella (AF), the undulating membrane (black asterisks), and axostyle (Ax) are not clearly distinguishable and appear adhered to the substrate (white arrowheads). A white asterisk indicates a parasite covered with HMDS film, resulting in the fusion of individual structures into a single continuous mass. (JPG) [file pone.0333745.s001.jpg]

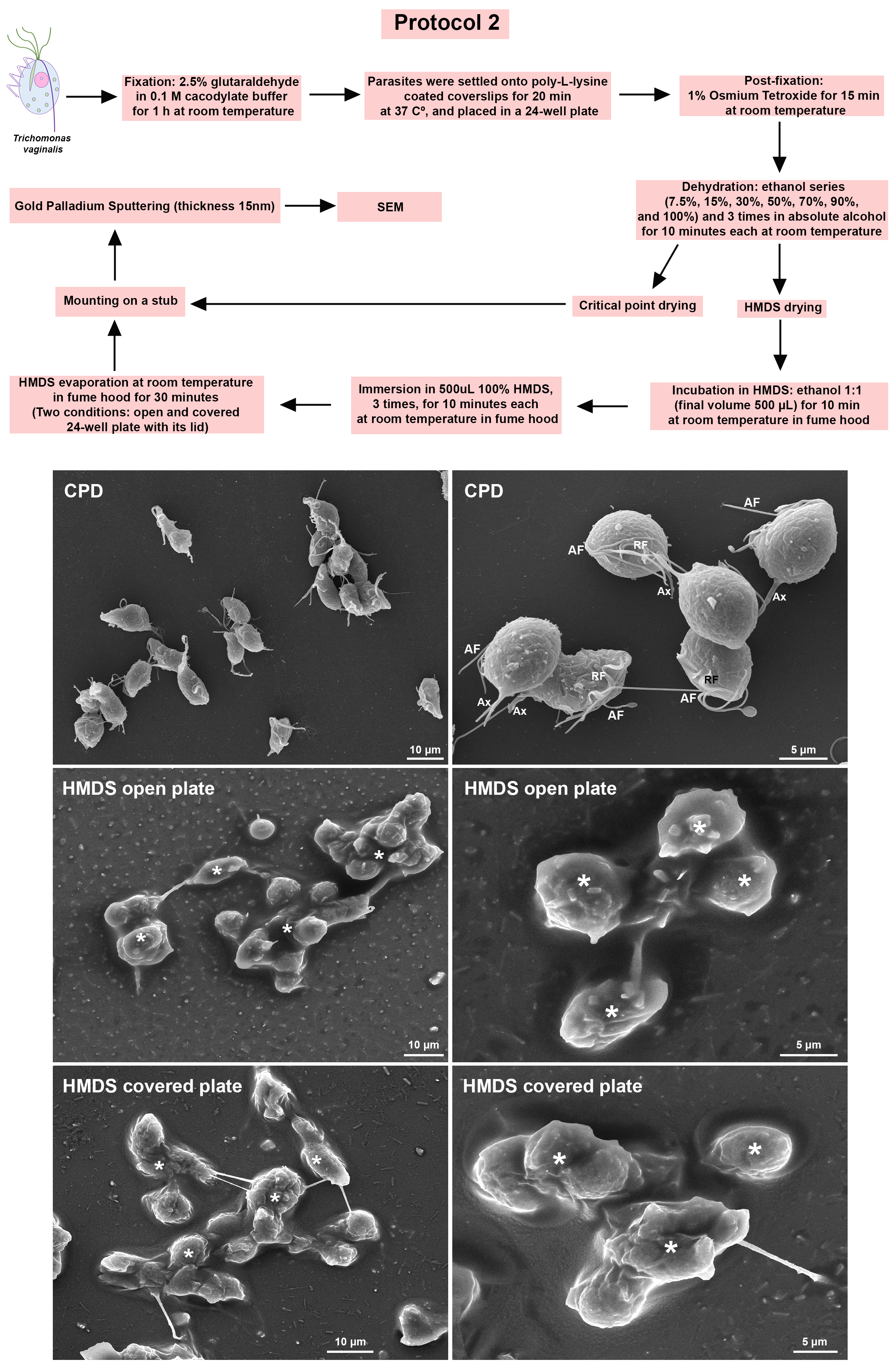

Supplement: S2 Fig — In CPD-prepared samples, parasites exhibit typical piriform or ellipsoid morphology with a slightly irregular surface displaying small undulations and microvesicles-like structures. Four anterior flagella (AF), a recurrent flagellum (RF), and the axostyle (Ax) tip in the posterior region of the parasite are clearly observed. In HMDS-prepared samples, parasites appear coated with a thick HMDS film, resulting in a wrinkled and shrunken surface and fusion of individual parasites into a single continuous mass, with a concomitant loss of distinct morphological features (*). (JPG) [file pone.0333745.s002.jpg]

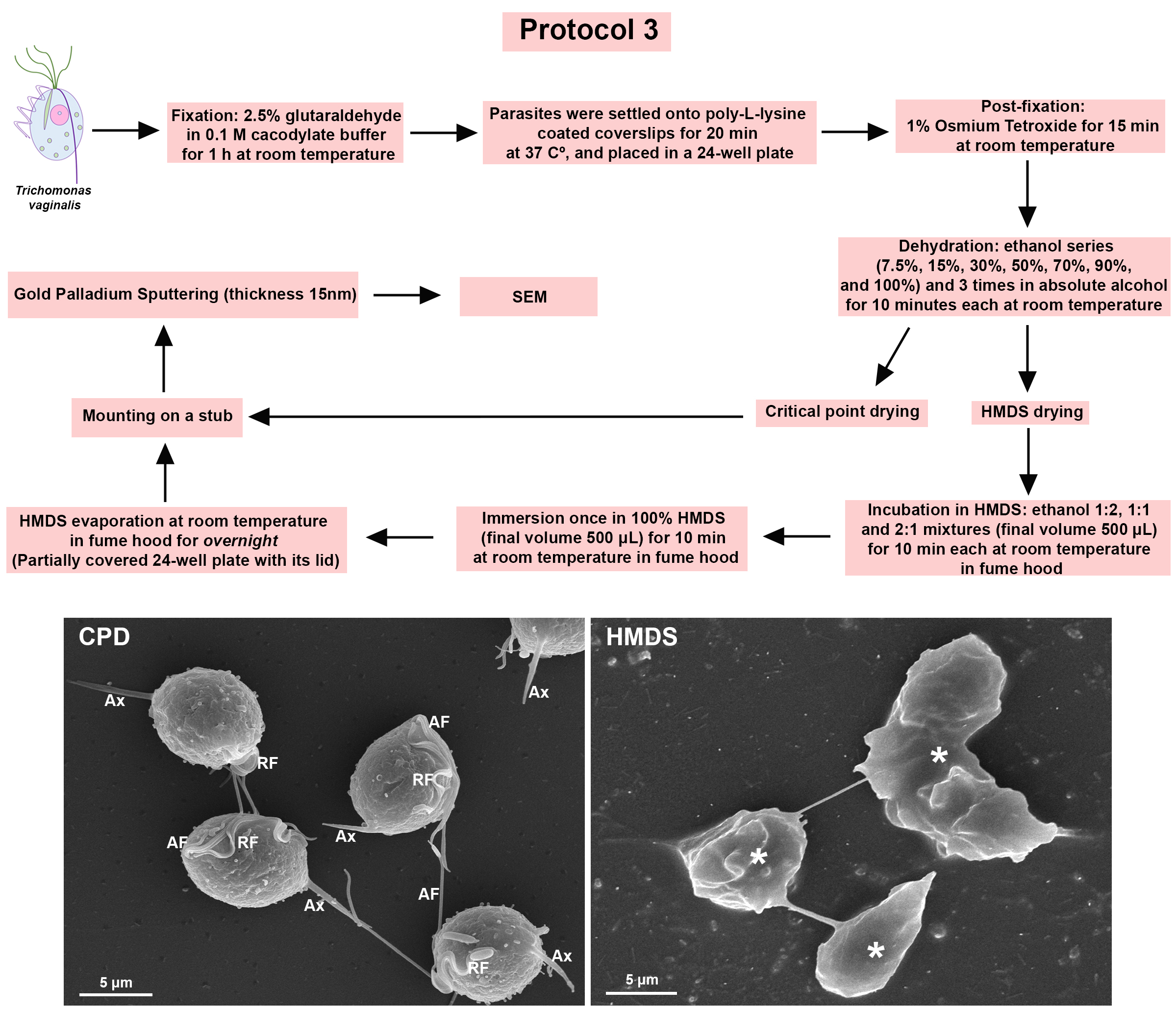

Supplement: S3 Fig — In CPD-prepared samples, parasites displaying typical piriform or ellipsoid morphology with a slightly irregular surface displaying small undulations and microvesicles-like structures. Four anterior flagella (AF), a recurrent flagellum (RF), and the axostyle (Ax) tip in the posterior region of the parasite are clearly observed. In HMDS-prepared samples, parasites appear coated with a thick HMDS film, resulting in a wrinkled and shrunken surface, and fusion of individual parasites into a single continuous mass, accompanied by the loss of distinct morphological features (*). (JPG) [file pone.0333745.s003.jpg]

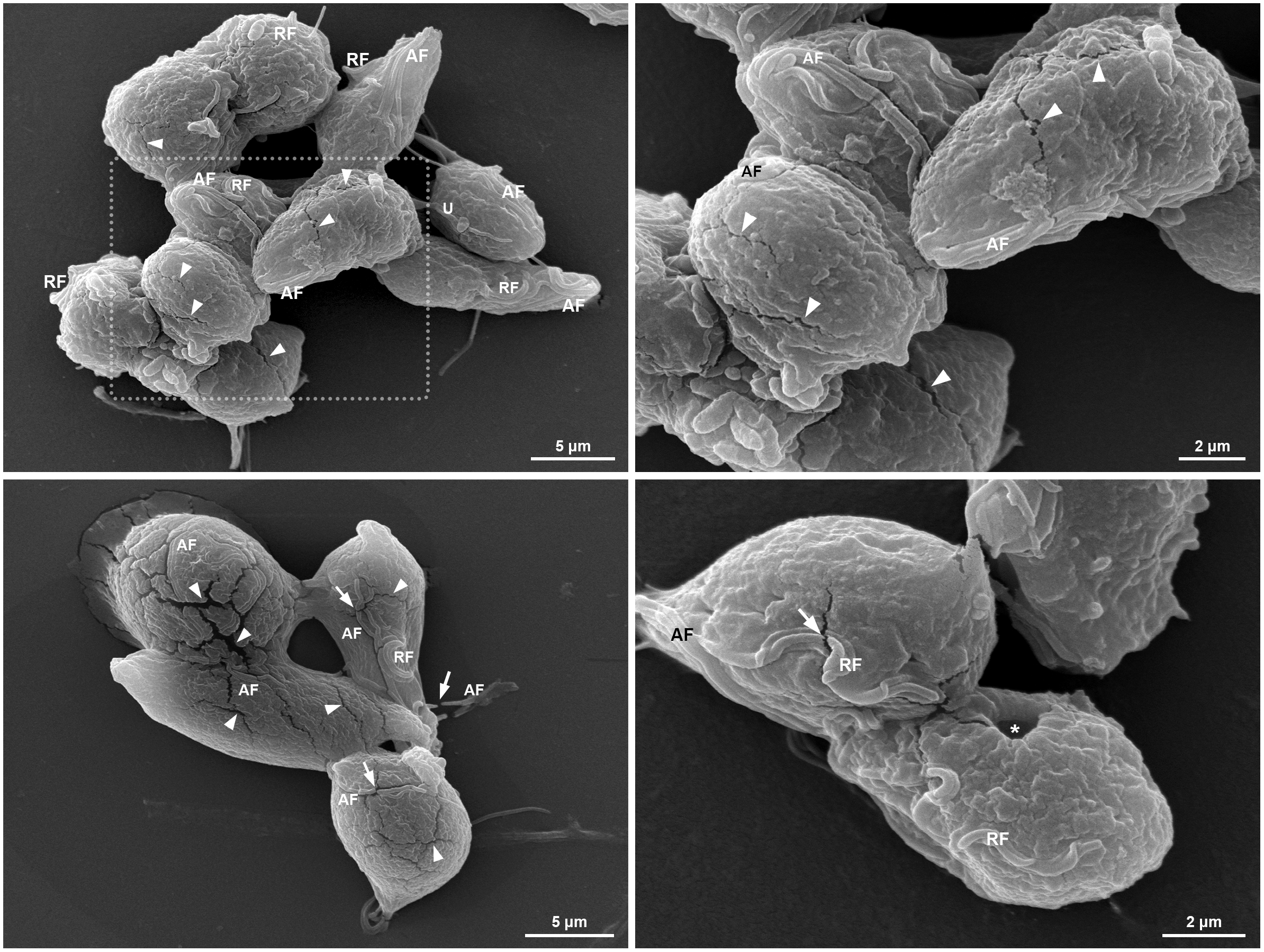

Supplement: S4 Fig — Morphological damage is evident in air-dried samples, including cell shrinkage, disrupted flagella (arrows), surface ruptures (arrowheads), and the presence of a perforation (*). AF, anterior flagella; RF, recurrent flagellum. (JPG) [file pone.0333745.s004.jpg]

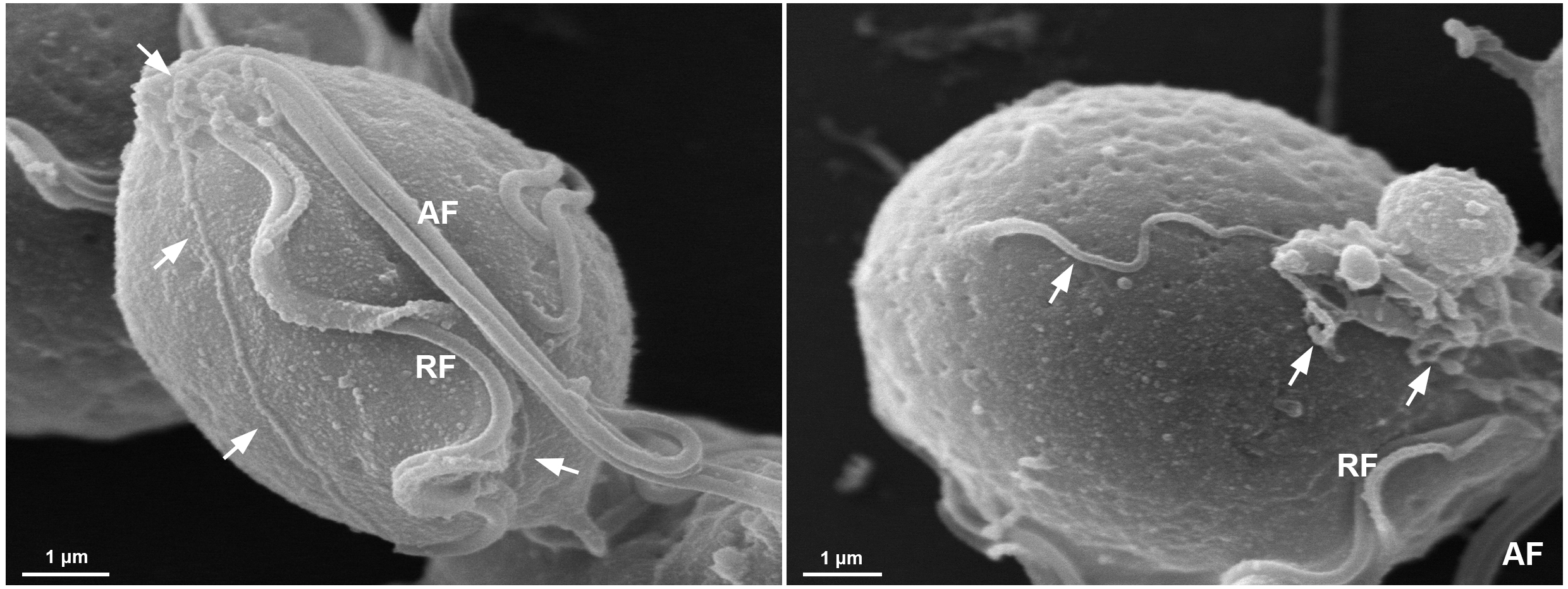

Supplement: S5 Fig — Worm-like structures (arrows) are observed on the cell body and at the flagellar base region of the parasites. AF, anterior flagella; RF, recurrent flagellum. (JPG) [file pone.0333745.s005.jpg]

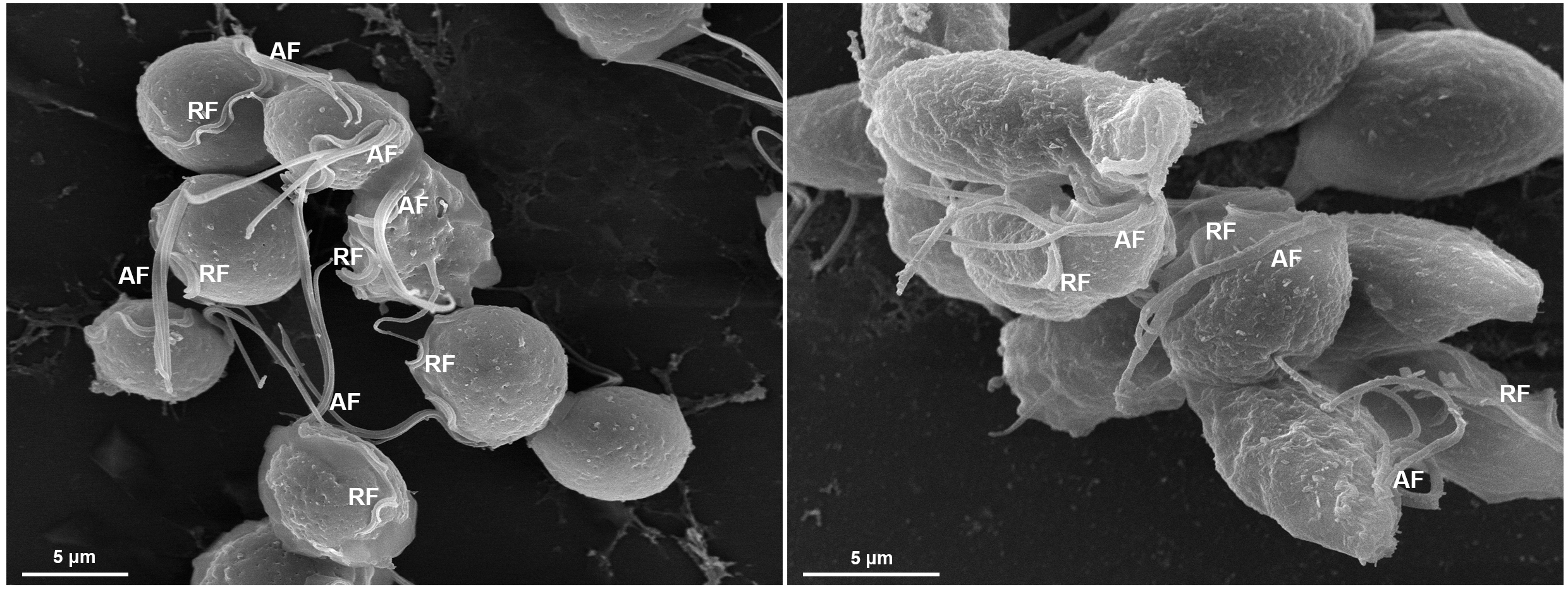

Supplement: S6 Fig — No surface projections are observed within the clumps. AF, anterior flagella; RF, recurrent flagellum. (JPG) [file pone.0333745.s006.jpg]

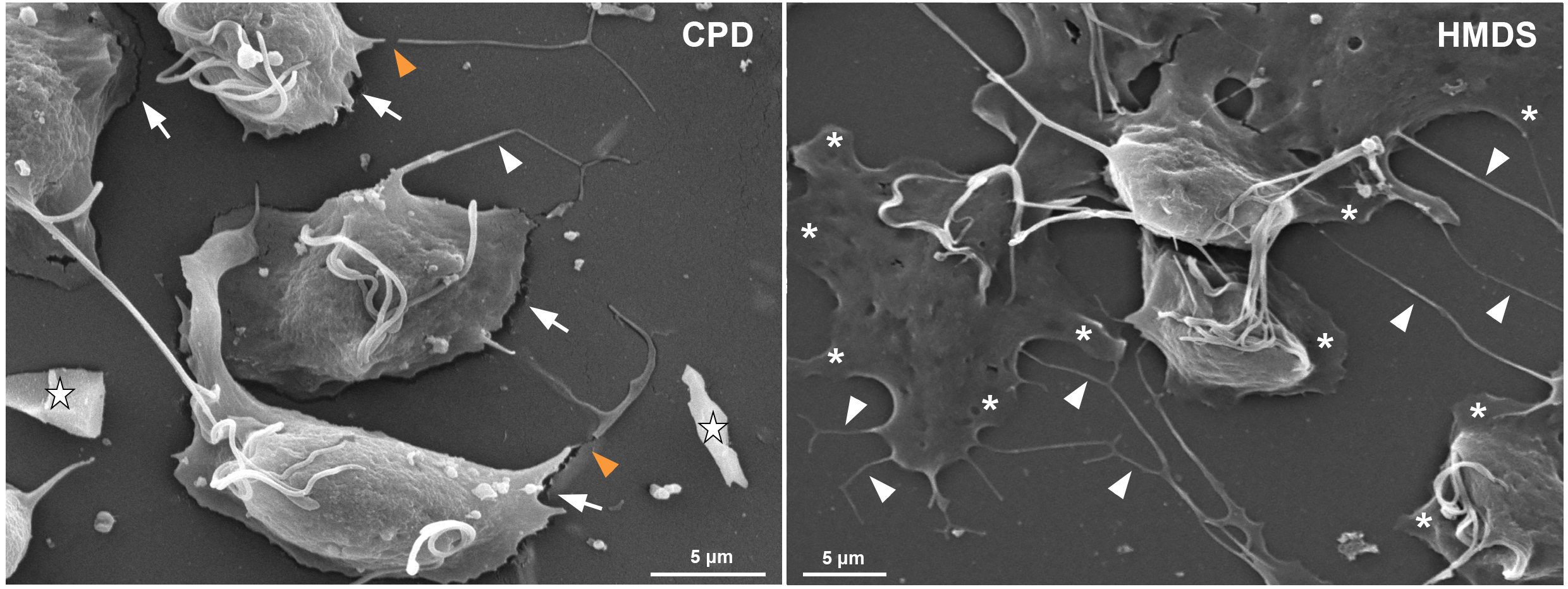

Supplement: S7 Fig — General and detailed views show notable differences in surface preservation between the two drying methods. In CPD-dried sample, lamellipodia and pseudopods appear retracted (white arrows), ruptured filopodia and cytonemes are observed (orange arrowheads) and fragments are seen (★). In contrast, HMDS-dried sample exhibit long, intact cytonemes connecting adjacent parasites, forming an intercellular network (white arrowheads). Broad, well-preserved lamellipodia and pseudopods are also seen spread across the substrate (*). (JPG) [file pone.0333745.s007.jpg]

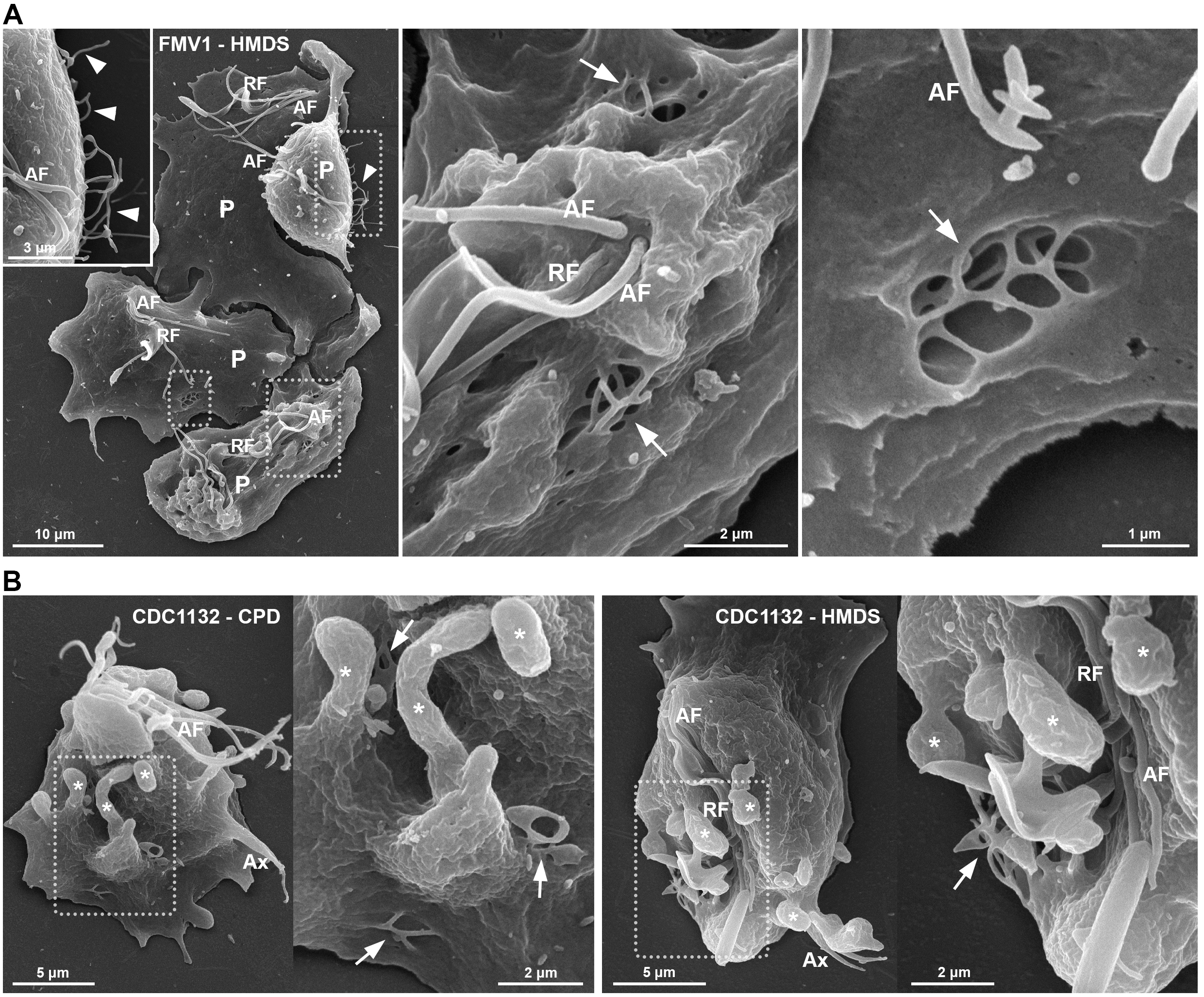

Supplement: S8 Fig — (A) HMDS-dried FMV1 parasites. (B) CPD- and HMDS-dried CDC1132 parasites. Mesh-like structures on the cell body are indicated by arrows. In (A), the inset shows cytonemes protruding from the cell body (arrowheads). In (B), pseudopods are indicated by asterisks. AF, anterior flagella; RF, recurrent flagellum. (JPG) [file pone.0333745.s008.jpg]
