# Supplementary material for: Replacing critical point drying with hexamethyldisilazane drying enhances the ultrastructural preservation of cell surface projections in the parasite Trichomonas vaginalis for scanning electron microscopy
Source: PLoS One. 2025 Oct 6;20(10):e0333745. doi: 10.1371/journal.pone.0333745 (PMC12500116; doi:10.1371/journal.pone.0333745)
Supplement: S1 Dataset — (DOCX) [file pone.0333745.s009.docx]

**Table A. Values used to build Fig 1D**

| Surface area of parasites (µm^2^) | | | | | |
| --- | --- | --- | --- | --- | --- |
| 1^st^  experiment | | **2^nd^ experiment** | | **3^rd^ experiment** | |
| CPD | **HMDS** | **CPD** | **HMDS** | **CPD** | **HMDS** |
| 35.722 | **55.130** | **43.144** | **47.917** | **79.184** | **101.588** |
| 30.806 | **21.459** | **78.041** | **99.886** | **67.407** | **84.049** |
| 43.583 | **53.619** | **64.794** | **80.158** | **77.362** | **98.875** |
| 66.784 | **55.191** | **56.718** | **68.132** | **74.656** | **94.845** |
| 88.331 | **54.995** | **29.909** | **28.206** | **56.672** | **68.063** |
| 31.586 | **59.752** | **29.907** | **28.204** | **76.294** | **97.285** |
| 62.361 | **98.591** | **23.977** | **20.123** | **25.819** | **22.116** |
| 31.338 | **54.575** | **72.921** | **92.261** | **32.329** | **31.811** |
| 52.889 | **52.810** | **56.860** | **68.353** | **23.198** | **20.124** |
| 71.414 | **70.403** | **63.345** | **78.000** | **40.163** | **43.478** |
| 43.963 | **55.917** | **21.706** | **20.123** | **44.000** | **49.192** |
| 44.470 | **60.449** | **79.204** | **101.618** | **36.894** | **38.609** |
| 71.955 | **79.938** | **70.878** | **89.218** | **70.653** | **88.884** |
| 51.106 | **51.919** | **33.320** | **33.286** | **42.067** | **46.312** |
| 51.816 | **58.759** | **31.472** | **30.534** | **37.474** | **39.473** |
| 46.443 | **57.791** | **31.567** | **30.676** | **53.329** | **63.084** |
| 50.256 | **56.741** | **38.886** | **41.576** | **28.994** | **26.845** |
| 42.948 | **38.633** | **52.242** | **61.466** | **69.046** | **86.490** |
| 51.975 | **34.330** | **46.621** | **53.094** | **24.974** | **20.858** |
| 50.917 | **55.743** | **38.098** | **40.402** | **80.232** | **103.149** |
| 47.777 | **87.438** | **57.517** | **69.322** | **67.232** | **83.789** |
| 43.787 | **46.530** | **28.908** | **26.716** | **32.495** | **32.057** |
| 51.765 | **84.343** | **38.153** | **40.485** | **20.794** | **20.125** |
| 54.145 | **66.285** | **42.649** | **47.179** | **69.849** | **87.687** |
| 57.555 | **66.052** | **48.082** | **55.270** | **63.271** | **77.891** |
| 65,189 | **50.816** | **68,015** | **84.955** | **64.613** | **79.889** |
| 48,094 | **54.926** | **32.553** | **32.144** | **67.173** | **83.701** |
| 57,801 | **59.848** | **51.605** | **60.517** | **24.944** | **20.812** |
| 44,731 | **63.669** | **56.340** | **67.568** | **42.170** | **46.467** |
| 59,371 | **65.549** | **23.272** | **20.123** | **27.477** | **24.585** |
| 52,233 | **51.184** | **57.256** | **68.933** | **72.735** | **91.984** |
| 55,323 | **34.377** | **30.787** | **29.515** | **58.210** | **70.354** |
| 55,590 | **64.657** | **24.399** | **20.128** | **40.501** | **43.980** |
| 40.851 | **40.468** | **77.930** | **99.721** | **24.309** | **20.123** |
| 50.253 | **52.280** | **78.945** | **101.232** | **39.294** | **42.184** |
| 50.905 | **46.115** | **69.421** | **87.05** | **40.154** | **43.465** |
| 37.348 | **63.765** | **38.909** | **41.609** | **64.649** | **79.943** |
| 39.456 | **44.809** | **26.375** | **22.944** | **59.074** | **71.640** |
| 59.109 | **62.125** | **61.901** | **75.850** | **74.195** | **94.159** |
| 91.600 | **72.731** | **47.118** | **53.835** | **49.060** | **56.727** |
| 35.595 | **42.866** | **27.851** | **25.141** | **27.703** | **24.921** |
| 30.301 | **55.827** | **50.450** | **58,798** | **63.658** | **78.467** |
| 22.987 | **107.537** | **22.542** | **20.126** | **66.538** | **82.755** |
| 43.412 | **51.477** | **75.534** | **96.153** | **54.454** | **64.760** |
| 30.153 | **48.414** | **36.133** | **37.475** | **67.154** | **83.673** |
| 44.385 | **48.662** | **60.586** | **73.892** | **50.367** | **58.673** |
| 36.466 | **41.858** | **39.338** | **42.249** | **52.119** | **61.283** |
| 46.781 | **45.430** | **51.958** | **61.043** | **46.354** | **52.697** |
| 33.576 | **40.527** | **53.572** | **63.446** | **21.999** | **20.127** |
| 39.656 | **50.403** | **31.655** | **30.807** | **26.994** | **23.865** |

**Table B. Values used to build Fig 2D**

|  | % parasites with cytonemes/filopodia | | | | | | | | | | | |
| --- | --- | --- | --- | --- | --- | --- | --- | --- | --- | --- | --- | --- |
| Strain | **1^st^  experiment** | | | | **2^nd^ experiment** | | | | **3^rd^ experiment** | | | |
|  | **CPD** | | **HMDS** | | **CPD** | | **HMDS** | | **CPD** | | **HMDS** | |
| FMV1 | **1.2** | **1.2** | **54.2** | **50.0** | **1.2** | **1.0** | **22.0** | **32.4** | **0.4** | **0.6** | **31.4** | **38.0** |
| CDC1132 | **42.0** | **48.0** | **48** | **46.0** | **53.0** | **62.0** | **64.0** | **56.0** | **59.8** | **42.8** | **62.8** | **56** |
| G3 | **1.0** | **0.8** | **0.8** | **0.6** | **0.6** | **1.0** | **0.8** | **1.0** | **0.4** | **0.4** | **0.4** | **0.4** |

**Table C. Length (µm) of 150 cytoneme-like projections in HMDS-dried FMV1 strain parasites**

| 30.056 | 0.887 | 0.673 | 3.715 | 0.604 | 0.935 | 8.222 | 0.618 | 2.113 | 13.269 | 3.187 | 0.229 | 1.152 | 0.639 | 3.106 |
| --- | --- | --- | --- | --- | --- | --- | --- | --- | --- | --- | --- | --- | --- | --- |
| 11.761 | 0.505 | 2.224 | 1.664 | 5.223 | 1.762 | 0.820 | 5.983 | 1.607 | 1.730 | 5.506 | 0.573 | 1.511 | 1.300 | 8.116 |
| 7.482 | 1.167 | 2.264 | 1.794 | 0.346 | 0.778 | 1.300 | 11.921 | 0.928 | 6.412 | 1.276 | 0.345 | 2.028 | 0.281 | 0.854 |
| 2.651 | 1.282 | 14.881 | 1.120 | 1.786 | 8.501 | 6.821 | 7.740 | 15.650 | 1.030 | 1.125 | 6.349 | 0.513 | 2.747 | 1.413 |
| 3.849 | 0.902 | 1.397 | 2.810 | 2.636 | 4.993 | 5.39 | 8.760 | 3.006 | 7.284 | 0.200 | 3.295 | 2.655 | 20.194 | 1.743 |
| 0.300 | 2.075 | 1.078 | 1.684 | 2.626 | 1.817 | 7.607 | 2.090 | 9.534 | 4.022 | 0.830 | 5.185 | 0.815 | 3.697 | 4.113 |
| 13.357 | 0.706 | 4.741 | 4.728 | 0.394 | 0.681 | 0.377 | 0.536 | 9.894 | 27.168 | 0.243 | 14.777 | 0.525 | 1.075 | 1.298 |
| 1.378 | 0.764 | 0.449 | 3.075 | 1.651 | 0.453 | 4.018 | 3.148 | 7.121 | 6.611 | 0.673 | 2.365 | 1.035 | 1.353 | 3.836 |
| 1.213 | 6.167 | 1.523 | 0.621 | 0.365 | 2.577 | 1.287 | 7.346 | 8.343 | 1.912 | 0.537 | 3.382 | 1.801 | 3.325 | 1.729 |
| 1.391 | 0.474 | 1.269 | 0.849 | 0.955 | 3.892 | 2.087 | 6.825 | 1.046 | 9.743 | 1.321 | 0.576 | 0.563 | 0.542 | 2.473 |

**Table D. Percentage of parasites with microvesicles on their cell surface. Values of FMV1 strain were used to build Fig 4B**

|  | % parasites with microvesicles | | | | | | | | | | | |
| --- | --- | --- | --- | --- | --- | --- | --- | --- | --- | --- | --- | --- |
| Strain | **1^st^  experiment** | | | | **2^nd^ experiment** | | | | **3^rd^ experiment** | | | |
|  | **CPD** | | **HMDS** | | **CPD** | | **HMDS** | | **CPD** | | **HMDS** | |
| FMV1 | **44.8** | **44.4** | **41.4** | **35.2** | **47.4** | **52.6** | **68.6** | **46.2** | **32.4** | **31.2** | **35** | **34.2** |
| CDC1132 | **34.8** | **29.6** | **26.6** | **34.0** | **39.6** | **37.2** | **31.4** | **32.6** | **45.0** | **41.4** | **38.6** | **34.6** |
| G3 | **10.2** | **10.4** | **10.4** | **9.0** | **8.0** | **7.0** | **9.2** | **6.2** | **12.2** | **12.0** | **13.8** | **12.4** |

**Tables E and F. Values used to build Fig 5B**

| % parasites with surface projections | | | | | | | | | | | |
| --- | --- | --- | --- | --- | --- | --- | --- | --- | --- | --- | --- |
| 1^st^  experiment | | | | **2^nd^ experiment** | | | | **3^rd^ experiment** | | | |
| CPD | | **HMDS** | | **CPD** | | **HMDS** | | **CPD** | | **HMDS** | |
| 34.2 | **32.6** | **94.6** | **95.6** | **29.4** | **29.4** | **90.8** | **87.4** | **22.0** | **27.2** | **85.8** | **90.0** |

| % parasites with disrupted projections | | | | | | | | | | | |
| --- | --- | --- | --- | --- | --- | --- | --- | --- | --- | --- | --- |
| 1^st^  experiment | | | | **2^nd^ experiment** | | | | **3^rd^ experiment** | | | |
| CPD | | **HMDS** | | **CPD** | | **HMDS** | | **CPD** | | **HMDS** | |
| 69.0 | **68.8** | **23.4** | **24.0** | **65.0** | **68.0** | **21.0** | **17.0** | **64.6** | **65.0** | **14.0** | **14.6** |
