# Supplementary material for: Replacing critical point drying with hexamethyldisilazane drying enhances the ultrastructural preservation of cell surface projections in the parasite Trichomonas vaginalis for scanning electron microscopy
Source: PLoS One. 2025 Oct 6;20(10):e0333745. doi: 10.1371/journal.pone.0333745 (PMC12500116; doi:10.1371/journal.pone.0333745)
Supplement: Attachment — Submitted filename: Response to Reviewers.docx [file pone.0333745.s012.docx]

**Response to Reviewers**

Ms. No. PONE-D-25-35035

Corresponding author: Antonio Pereira-Neves

Manuscript title: Replacing critical point drying with hexamethyldisilazane drying enhances the ultrastructural preservation of cell surface projections in the parasite *Trichomonas vaginalis* for scanning electron microscopy

Editor Comments:

I am pleased to inform you that one reviewer already accepted the revised manuscript for publication and the other reviewer (reviewer 3) requested some revisions for future publication endorsement. Please carefully answer reviewer 3' concerns and rectify the manuscript following his/her comments. Thank you for choosing PLOS ONE journal and best regards,

António Machado

Dear Dr. Machado, Academic Editor of PLOS ONE

Initially, we would like to thank you for handling our manuscript and for your thoughtful update on the review process and the Reviewers for their valuable time and comments which definitely contributed to improve the manuscript. We are very pleased to learn that one of the reviewers has already endorsed our revised submission, and we greatly appreciate Reviewer 3’s constructive feedback.

As can be seen below, we carefully addressed all of his/her concerns and revised the manuscript accordingly to ensure it meets the journal’s standards. The alterations are highlighted in the file with the track changes. We believe that the manuscript has had improvement.

Thank you once again for your efforts on our behalf, and for considering our work for publication in *PLOS ONE*.

Response to Reviewers

*We are grateful to the reviewers for the time and dedication invested in carefully reading and evaluating our manuscript. We very much appreciate the positive remarks regarding the experimental design, presentation of the results, and the overall clarity and informative value of our study.*

**Response to Reviewer #1**

*We are sincerely grateful for the reviewer’s detailed and encouraging evaluation of our manuscript. Her/his generous compliments regarding the clarity and technical quality of our work are deeply appreciated.*

*We also thank the reviewer for sharing her/his own experiences with HMDS. We are encouraged to know that our findings may contribute to improving specimen preparation practices in other laboratories.*

*Importantly, we are very grateful for the suggestion to employ cryo-SEM in future studies. We fully agree that cryo-SEM has strong potential to preserve surface appendages of T. vaginalis in their most native state, free of chemical or physical drying artifacts, and we will certainly consider this powerful approach to complement and expand upon our current work.*

*Once again, we thank the reviewer for her/his thoughtful assessment and generous words, which serve as great motivation for our ongoing and future investigations.*

**Response to Reviewer #3**

This paper by dos Santos Melo and colleagues was an interesting read. The differences between the present study’s approach and that of a previous study (Malli and colleagues 2018) is clearly discussed (as it should be, given the shared context between it and the present manuscript), although it is a bit odd this is primarily covered at the beginning of the results section rather than the Introduction. Actually, my impression was that the end of the Introduction itself could be improved regarding clarity and structure; the Introduction reasonably overviewed Trichomonas vaginalis, its established relevance as a sexually transmitted pathogen, and reasonably overviewed SEM/CPD and potential issues with it and why HMDS drying might be preferable (in a broad sense). However, the connection of these overviews to the core focus of the paper (application of HMDS incubation vs CPD to T. vaginalis compared against the results of the prior study by Malli et al. 2018) could have been more smoothly done towards the end of the Introduction. However, the beginning of the Results section resolves this issue quite well, so I don’t want to complain about it too much. As is, the paper’s writing and structuring works well enough for the interested reader to follow it. The results and discussion sections were particularly informative, and, in my opinion, adequate checks/controls were performed to ensure the observed differences in cell surface morphology did not originate from experimental artifacts. Testing not only the FMV1 strain but also the known highly adherent CDC1132 and known poorly adherent G3 strains with both the HMDS and CPD protocols was a good decision in experimental design to ensure the observed cytoneme-like structures and extracellular vesicles (including microvesicles) in the HMDS FMV1 strain cells were not induced via exposure to HMDS. Additionally, the hypothesized explanation by the authors for why the FMV1 strain cell morphology responded so differently to preparation via CPD vs HMDS incubation was interesting and certainly not implausible. The authors don’t stop here, however; they then go and apply their modified HMDS drying protocol to observe T. vaginalis inter-cell interactions via filipodia, particularly via the cytonemes. Again, very appropriate experimental design in my opinion, and it led to an enjoyable read of this manuscript. Actually, the authors apply their modified HMDS protocol to the study of T. vaginalis cells in quite a few ways. This was unexpected and I don’t think strictly necessary for the publication of this manuscript (honestly I think it could almost be two separate, smaller papers), but it’s certainly not a problem. Seeing the structures observed on the amoeboid forms of the pathogen, as well as with the interaction of the pathogen with host prostate cells was interesting and certainly helps support the broad application/robustness of their modified HMDS sample drying approach in application to the T. vaginalis pathogen. Also, to be clear, my expertise relevant to this manuscript is more so regarding the SEM side of things; I certainly have a background in molecular/cell biology and microbiology, but I do not regularly research microbes or pathogens.

I will note, it would be nice if the underlying mechanisms explaining why the cells responded the way they did to HMDS incubation were able to be presented. It’s certainly fine they aren’t, but as is, this manuscript leans more towards a microbiology-heavy paper rather than an SEM protocol optimization paper applied to a microbiological context. The paper title and the Introduction sections imply a bit more of an emphasis on the SEM protocol optimization side of things, so this was initially a bit unexpected. However, again, the paper does work well for what it is, and I would not be too concerned over this. The way it’s written, it’s very informative; almost like a review article containing an SEM sample preparation protocol optimization procedure within it.

*We very much appreciate the positive remarks regarding the experimental design, presentation of the results, and the overall clarity and informative value of our study. We are also very appreciative of the reviewer’s detailed and constructive input, which helped us strengthen the manuscript and enhance its readability. We would particularly like to highlight our gratitude for the reviewer’s detailed comments on wording and phrasing. As non-native English speakers, we recognize the importance of these clarifications to ensure that the text remains precise, balanced, and accessible to a broad readership. The reviewer’s suggestions had greatly assisted us in refining the manuscript improving clarity. Certainly, we intend to investigate why the cells responded the way they did to HMDS incubation, but as raised by the reviewer, this topic leans more towards a microbiology-heavy paper rather than an SEM protocol optimization paper applied to a microbiological context.*

*We agreed and incorporated the reviewer’s suggestions. The Introduction section was slightly reorganized: the first and second paragraphs of the Results and Discussion were moved to the Introduction, and the final paragraph was modified to better balance the emphasis on both technical and biological insights. We believe this restructuring ensures a clearer narrative progression, moving from the importance of the parasite → role of surface structures → SEM use and challenges → CPD vs. HMDS drying → rationale of our study.*

The comments below are all relatively minor, although I will note they apply mostly to the Introduction. The writing of the Results and Discussion sections was generally more appropriate than that of the Introduction. There are places in the Introduction section where the authors do need to be more balanced/measured in the information they present:

Line 44 replace the word “confirms” with perhaps “demonstrates” or something else; “confirms” is too close to “proves”, and obviously nothing is 100% proven within empirical science *Thank you for the correction. It was replaced*

Line 51 What makes the pathogen overlooked?

*Thanks for the question. Despite being the most common non-viral sexually transmitted infectious agent (STIA) worldwid****e****, Trichomonas vaginalis has historically been regarded as a “minor” STIA compared to HIV, syphilis, or gonorrhea, largely due to asymptomatic cases, diagnostic challenges, and the perception of being a less severe infectious agent. This perception contributes to limited attention from public health agencies and funding bodies. Funding for research, surveillance, and drug development is disproportionately low compared to its prevalence. Standard diagnostic tools, such as wet mount microscopy, have low sensitivity and lead to frequent underdiagnosis, while the lack of mandatory reporting in many regions results in less robust epidemiological data. Moreover, men are rarely screened, obscuring the true burden of infection. The association of trichomoniasis primarily with women’s reproductive health reinforces stigma while diverting attention from male carriers, who are equally important in transmission. There is also limited innovation in treatment (metronidazole and tinidazole remain the only mainstays, despite emerging resistance).*

*For the reasons outlined above, some authors have described T. vaginalis as an ‘overlooked’ pathogen. To present the information in a more balanced manner, we replaced the term ‘overlooked’ with ‘neglected’.*

Lines 61-62 Statement needs to be cited, don’t just blanket claim many aspects of the pathogen cell biology and pathogenicity remain unknown or understudied. *Thanks for the correction. We included the following statement: “e.g., role of extracellular vesicles and surface protrusions in parasite-to-parasite and parasite-to-host cell interaction, strain variability, and interaction with host microbiome and immunity.*

Line 67 “undoubtedly a powerful tool” change to perhaps “useful tool” especially the word “undoubtedly”; I agree SEM is a useful tool for study cell structure, but more measured wording would be more appropriate and less polarizing. *Many thanks for the suggestion. We changed* *“undoubtedly a powerful tool” to “useful tool”.*

Line 76 point out that the need for some sort of sample processing is common for most biological specimens, so readers unfamiliar with biological SEM understand this isn’t strictly limited to T. vaginalis surface structure.

Line 76 I would change wording to “it generally requires meticulous processing” to be more measured; there are cases in which essentially unprepared biological samples can observed with SEM; for example, see environmental SEM

Lines 76-80 environmental SEM does exist, with which fully hydrated specimens can be observed using SEM; thus water removal is not always necessary

*We appreciated and are thankful for these reviewer’s suggestions. Accordingly, the sentences were revised, and the information is now restricted to high-vacuum SEM. The updated version reads as follows: “However, visualizing these structures with a conventional high-vacuum SEM is not a trivial task; as common for most biological specimens, it generally requires meticulous processing to accurately preserve ultrastructural details as close as possible to their natural state. That involves cell fixation, dehydration in an intermediate solvent, such as ethanol or acetone, drying and sputter coating. A crucial challenge in preparing biological specimens for high-vacuum SEM is water removal.”*

Line 81 “by far” again, I would change the wording to be a bit more measured, maybe say “is generally the most commonly used method”. *Thanks for the suggestion.* *We changed* *“by far” to “ is generally the most commonly used method”.*

Line 87 change “Although CPD is considered the state-of-the-art drying method” to “Despite the wide usage of the CPD drying method” or something comparable; as is, saying CPD drying is state-of-the-art is a strong assumption, and I’m not sure every SEM expert would refer to it in that manner. I certainly wouldn’t refer to it in that manner.

Lines 96-98 see, here it’s stated that hexamethyldisilazane has been used before as an alternative to CPD, so again, referring to CPD as state-of-the-art is a very arguable statement

*We appreciate and are thankful for these reviewer’s suggestions. We agreed and changed “Although CPD is considered the state-of-the-art drying method” to “Despite the wide usage of the CPD drying method”.*

Line 92 change “longer” to “substantial”, the word “longer” implies CPD is being compared to something, and that isn’t the case yet at this point in the Introduction. *Thanks for the correction. We changed “longer” to “substantial”.*

Lines 94-95 use more measured wording here than “highly recommended”, such as “may be preferable”; you’ve presented the evidence for your argument, try to avoid strongly pushing the reader towards thinking a specific way; let the reader decide for themselves.

Line 95 add on “such as those of the T. vaginalis cell surface.” or something comparable at the end of this sentence on line 95 to help relate this SEM theory to your target pathogen

*Thanks for these suggestions. We changed “highly recommended” to “may be preferable” and added “such as those of the T. vaginalis cell surface” at the end of sentence .*

Line 112 wording must be changed to “that some highly adherent strains” because the very next sentence states that some other researchers have observed mixed numbers of cytoneme-like structures in some highly adherent strains; so clearly elevated numbers of cytoneme-like structures have not yet been observed for all strains of T. vaginalis; without this change in wording, these sentences are confusing and read as if they contradict themselves. *Thanks for the correction. We changed to “that some highly adherent strains”*

Lines 362-368 would be helpful if these definitions for “filipodia” and “cytonemes” in context of T. vaginalis were presented earlier in the manuscript, given these terms are used quite a bit prior to their being defined here. *Thanks for the suggestion. Now, those definitions are presented earlier in lines 230-235*

Line 668 maybe use “affirms” instead of “confirms”; again, “confirms” is too close to “proves”. Thanks for the correction. We changed “confirms” to “affirms”
